# Robustness of spatial learning in flickering networks

**DOI:** 10.1186/1471-2202-16-S1-P43

**Published:** 2015-12-18

**Authors:** Yuri A Dabaghian, Samir Chowdhury, Andrey Babichev, Facundo Mémoli

**Affiliations:** 1 Neurology-Pediatrics Department, Baylor College of Medicine, Houston, TX 77030, USA; 2 Computational and Applied Mathematics, Rice University, Houston, TX, 77005, USA; 3 Department of Mathematics, Ohio State University, Columbus, OH, 43210, USA

## 

It is widely accepted that the network of the hippocampal place cells provides a substrate of the "cognitive map" of the environment. However, thousands of hippocampal neurons die every day and the networks formed by these cells constantly change due to various forms of synaptic plasticity. What then explains the remarkable reliability of our spatial memories? We propose a computational approach to answering this question based on a couple of insights. First, we propose that the hippocampal cognitive map is fundamentally topological, i.e., more similar to a subway map than to a topographical city map [1], and hence it is amenable to analysis by topological methods [2,3]. We then apply several novel methods from homology theory, to understand how dynamic connections between cells influences the speed and reliability of spatial learning. We simulate the rat's exploratory movements through different environments and study how topological invariants (stable topological features of different experimental environments) arise in a network of simulated neurons with dynamic, "flickering" connectivity. We find that despite transient connectivity the network of place cells produces a stable representation of the topology of the environment.
